# Insular and occipital changes in visual snow syndrome: a BOLD fMRI and MRS study

**DOI:** 10.1002/acn3.50986

**Published:** 2020-03-10

**Authors:** Francesca Puledda, Dominic Ffytche, David J. Lythgoe, Owen O’Daly, Christoph Schankin, Steven C. R. Williams, Peter J. Goadsby

**Affiliations:** ^1^ Headache Group Department of Basic and Clinical Neuroscience Institute of Psychiatry, Psychology & Neuroscience King’s College London London United Kingdom; ^2^ NIHR‐Wellcome Trust King’s Clinical Research Facility King’s College Hospital London United Kingdom; ^3^ Department of Old Age Psychiatry Institute of Psychiatry, Psychology & Neuroscience King’s College London London United Kingdom; ^4^ Centre for Neuroimaging Sciences Department of Neuroimaging Institute of Psychiatry, Psychology & Neuroscience King’s College London London United Kingdom; ^5^ Department of Neurology, Inselspital Bern University Hospital University of Bern Bern Switzerland

## Abstract

**Objective:**

To investigate the pathophysiology of visual snow (VS), through a combined functional neuroimaging and magnetic resonance spectroscopy (^1^H‐MRS) approach.

**Methods:**

We applied a functional MRI block‐design protocol studying the responses to a visual stimulation mimicking VS, in combination with ^1^H‐MRS over the right lingual gyrus, in 24 patients with VS compared to an equal number of age‐ and gender‐matched healthy controls.

**Results:**

We found reduced BOLD responses to the visual stimulus with respect to baseline in VS patients compared to controls, in the left (*k* = 291; *P* = 0.025; peak MNI coordinate [‐34 12 ‐6]) and right (*k* = 100; *P* = 0.003; peak MNI coordinate [44 14 ‐2]) anterior insula. Our spectroscopy analysis revealed a significant increase in lactate concentrations in patients with respect to controls (0.66 ± 0.9 mmol/L vs. 0.07 ± 0.2 mmol/L; *P* < 0.001) in the right lingual gyrus. In this area, there was a significant negative correlation between lactate concentrations and BOLD responses to visual stimulation (*P* = 0.004; *r* = −0.42), which was dependent on belonging to the patient group.

**Interpretation:**

As shown by our BOLD analysis, VS is characterized by a difference in bilateral insular responses to a visual stimulus mimicking VS itself, which could be due to disruptions within the salience network. Our results also suggest that patients with VS have a localized disturbance in extrastriate anaerobic metabolism, which may in turn cause a decreased metabolic reserve for the regular processing of visual stimuli.

## Introduction

Visual snow (VS) is a neurological condition characterized by the constant, panfield perception of small flickering dots.^1^ In the VS syndrome, patients also experience a combination of palinopsia, photophobia, entoptic phenomena, and nyctalopia.^2^ VS constitutes a spectrum type disorder that can be worsened by its most common comorbidities, migraine, and tinnitus. Hallucinogenic persisting perceptual disorder^3^, ^4^ can sometimes mimic VS, although the two are certainly separate conditions.^5^

VS pathophysiology is currently unknown; hypotheses include thalamo‐cortical dysrhythmia of the visual pathways,^6^ hyperexcitation of primary and secondary visual cortices,^7^, ^8^, ^9^ increased saliency of normally ignored subcortical activity or perhaps a combination of all these mechanisms.^10^ In particular, evidence for dysfunctional visual processing within the association cortices has emerged in VS, through the use of visual‐evoked potentials.^7^ An [^18^F]‐FDG PET study showed increased metabolism in the right lingual gyrus of VS patients compared to controls, implying a direct involvement of this area in VS pathophysiology.^11^ The lingual gyrus is a key associative visual processing region, containing lower‐level topographic representation of the contralateral visual field posteriorly, and higher‐level visual processing anteriorly, as it merges with the retrosplenial cortex and parahippocampal gyrus.^12^ Interestingly, the lingual gyrus is also known to be involved in migrainous photophobia,^13^, ^14^ a symptom that, without pain, is also present in VS.

Here we investigated cortical blood oxygenation level‐dependent (BOLD) responses to visual stimulation in patients with VS syndrome compared to healthy volunteers, through the use of a functional magnetic resonance imaging (fMRI) experiment in which subjects were shown a visual task mimicking the effect of the “visual snow” itself. We chose this particular stimulus in order to investigate differences in the patient population between an external and an internally perceived snow, and also to evaluate the effects of VS‐like stimulus on healthy individuals.

We also studied the neurochemical properties of the lingual gyrus in VS patients, through proton magnetic resonance spectroscopy (^1^H‐MRS). MRS provides a flexible and noninvasive way to quantify tissue metabolism directly.^15^ In particular, ^1^H‐MRS allows measurement of the concentrations of important brain metabolites such as choline, N‐acetylaspartate (NAA), glutamate, and lactate.

Our ultimate aim was to understand more about the underlying biology of VS. On the basis of previous neurophysiological and neuroimaging findings in this condition, we hypothesized that the lingual gyrus would show a dysfunctional energy metabolism, and that the visual association cortices would present an abnormal response to visual stimulation, in VS patients.

## Methods

### Subject population and recruitment

Twenty‐four patients with a diagnosis of VS syndrome according to the current criteria^2^ and an equal number of age‐ and gender‐matched healthy volunteers were selected for the study. This number of subjects per group was chosen based on the current literature.^16^ We recruited patients by email, re‐approaching subjects who had previously contacted our study team asking to participate in research studies. Healthy volunteers were recruited through internal advertisement at King’s College London.

Recruitment was limited to individuals of 20–60 years of age with no contraindications to MRI, no serious medical conditions and who were naïve to any type of recreational drugs, including cannabis. This was in order to exclude any possible misdiagnosis or overlap with hallucinogen persisting perception disorder^4^ and to allow homogeneity between the two subject cohorts. Any participant taking recurrent medications with an action on the central nervous system was excluded from the study. Patients with a history of psychosis, depression or psychological diseases either requiring ongoing psychoactive drugs, or drugs that were thought likely to affect the patient’s neural pathways, were excluded from the study. Volunteers were selected based on matching the age (±5 years) and gender of our patient population. All controls with a history of migraine or recurrent headaches were excluded.

All participants gave their informed consent. The study was approved by the London ‐ City & East Research Ethics Committee (Reference number: 16/LO/0964).

### Study protocol

The study involved a telephone interview, in which eligibility of the participant was assessed, followed by either one or two visits to our research facility, depending on whether the subject was in the patient or control group. During the first visit, patients underwent a full medical history taking, as well as a general examination and a neurological examination, blood pressure, and heart rate monitoring. If participants were deemed eligible, they were invited for a second visit in which the scanning took place, that lasted approximately 70 min.

All participants were scanned at the same time of day (between 9 and 12 am). Subjects were instructed to consume a light breakfast and to avoid caffeine prior to the visit. Participants were asked to refrain from the use of any type of medication for 24 h prior to scanning. If this was not avoidable, the scanning visit was postponed. Female patients were asked to keep a menstruation diary for the time of the study, in order to avoid scanning on days of active menstruation.

### Magnetic resonance imaging

All scans were conducted on a 3T General Electric MR750 MRI scanner at the NIHR‐Wellcome Trust King’s Clinical Research Facility, King’s College Hospital, London using a 12‐channel head coil. The scanning protocol was the same for both groups and was conducted over a single session. High‐resolution 3D T1‐weighted IR‐SPGR images were acquired. The parameters of this scan were: TR = 7.312 msec; TE = 3.016 msec; TI = 400 msec; flip angle = 11°; FOV = 270 mm; matrix = 256×256; slice thickness = 1.2 mm; 196 slice partitions, ASSET factor = 1.75, in‐plane resolution = 1 mm.^17^

Functional magnetic resonance echo‐planar images had the following parameters: TR = 2000 msec, TE = 28 msec, flip angle = 75°, 64 × 64 matrix. Each whole‐brain image contained 38.3 mm axial slices with a gap of 0.3 mm and 192 time points.

### BOLD fMRI

Blood oxygenation level‐dependent (BOLD) activity was quantified with a single fMRI experiment, characterized by a block‐design paradigm of visual stimulation and rest blocks of 40 sec each. Participants were presented with either a dark screen or a simulation of VS and were asked to keep their eyes open for the duration of the entire fMRI experiment. The onset of visual stimulation was triggered by the start of the scan acquisition.

**Figure 1:**
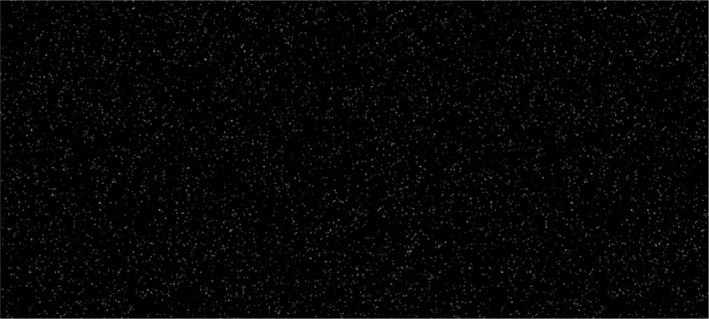
An image of the visual snow simulation video used for the fMRI experiment.

The simulation video mimicking the static flickering pattern typical of VS was developed by creating 20 bitmaps that were displayed in a continuous loop with 20 msec between frames. Each bitmap was created as random “snow squares” on a black background. Snow squares were blocks of 2 × 2 pixels. Five values of grayness were used to create these squares. These were 40, 80, 120, 160, and 200 where 0 = black, 255 = white. For each grayness value, two snow squares were created. These snow squares were randomly distributed within the black background in the ratio of 10:200 snow squares to background squares. An image of the simulation video is shown in Figure 1.

### Image processing and analysis

All fMRI data were processed and analyzed using the Statistical Parametric Mapping software suite, version 12 (SPM 12; http://www.fil.ion.ucl.ac.uk/spm/) in MATLAB R2017a (https://uk.mathworks.com/). Data preprocessing included manual reorientation to the anterior commissure, coregistration and image realignment, spatial normalization via unified segmentation into Montreal Neurological Institute (MNI) stereotactic space, and spatial smoothing (FWHM 8 mm).

First‐level voxel‐wise analysis was performed using a general linear modeling approach based on subject‐specific responses for the visual task. Each participant’s head movements were modeled as nuisance regressors. A regressor encoding the blocks of task‐stimulus was convolved with the haemodynamic response function, with the blank screen condition left un‐coded to serve as an implicit baseline. *T*‐statistic maps were calculated for the parameter estimates (beta values) of the main stimulus regressor. The resultant parameter estimates for the conditions of interest (increases and decreases in BOLD signal) were taken forward to a whole‐brain random‐effects analysis to test for the effect of task (blank screen vs. visual stimulus) and group (patients vs. controls). *Z* (Gaussianized *t*) statistic images were reviewed with an initial cluster‐forming voxel threshold of *P* < 0.001 and were family‐wise error (FWE) corrected, on the basis of cluster extent, to *P* < 0.05, using the Gaussian random field theory.^18^ The following variables: subject age, gender, handedness, migraine, and tinnitus presence were added as covariates in the model.

### Magnetic resonance spectroscopy

Proton spectra were acquired using a point resolved spectroscopy (PRESS) protocol with chemically selective suppression (CHESS) water suppression,^19^ while patients were at rest. A 1.5 × 3 × 1.5 cm^3^ voxel was placed over the right lingual gyrus. Anatomically, this was defined by identifying the area between the calcarine and collateral fissures, as anterior as possible in order to avoid areas of exclusive retinotopic representation. Voxel location was adjusted for each participant to maximize gray matter (GM) content. T1‐weighted images were used for voxel placement and for tissue segmentation. Prior to collection of MRS data, an automated prescan was performed to optimize transmit and receive gains and to optimize linear shimming gradients to improve homogeneity of the magnetic field within the voxel. Acquisition parameters were: TR = 3000 msec, TE = 30 msec, number of averages = 96, bandwidth = 5 kHz, number of points = 4096. Unsuppressed water reference spectra (16 averages) were also acquired. The total acquisition time was six minutes.

MRS data were processed using Linear Combination Model (LCModel version 6.3‐1L),^20^, ^21^ using an experimentally acquired basis set, acquired at the same field strength and echo time as the *in vivo* data, to calculate water‐scaled metabolite concentrations. The structural 3D IR‐SPGR was segmented into gray matter, white matter (WM) and cerebrospinal fluid (CSF) fractions using a Matlab script and SPM‐12, by creating an image of the MRS voxel in the same coordinate space as the structural image and subsequently calculating the averages of the GM/WM/CSF fractions in the 3D GM/WM/CSF images that lie within the MRS voxel.

Each individual's metabolite concentrations were corrected for partial volume confounds and differing amounts of water in each tissue type with the following formula:

$$\begin{array}{cc} & {\text{Metabolite}}_{\text{corrected}}\\ & \;=\frac{{\text{Metabolite}}_{\text{raw}}*\left(43300*f_{\text{GM}}+35880*f_{\text{WM}}+55556*f_{\text{CSF}}\right)}{35880*\left(1-f_{\text{CSF}}\right)} \end{array}$$

in which 43,300, 35,880, and 55,556 mmol/L are the water concentrations for GM, WM, and CSF, respectively. Metabolite_corrected_ represents the metabolite concentration from the gray and white matter proportion of the MRS voxel. The numerator corrects for differing tissue water concentrations for the unsuppressed water reference, whereas the denominator corrects for assumption that CSF is free of metabolites. The additional factor of 35,880 in the denominator is applied because the default LC model analysis assumes the voxel is pure white matter. Apart from assuming T_2_ = 80 msec for tissue water, no further corrections were applied for metabolite and tissue T_1_ and T_2_ relaxation.

The spectra signal‐to‐noise ratio, line width, and Cramér Rao lower bound (CRLB) error estimates (for each metabolite) reported by LC model were used as quality control measures. LC model fitted spectra were visually inspected for artifacts. Spectra with linewidth >0.067 ppm (8.5 Hz) and signal‐to‐noise (S/N) ratio <12 were excluded from further analysis.

### Correlation analysis

An anatomical region‐of‐interest (ROI) was created for the right lingual gyrus using the “wfu_pickupatlas Anatomical Library” (https://www.nitrc.org/projects/wfu_pickatlas/) within SPM. This area largely corresponded to the spectroscopy voxel of interest. On the basis of the *a priori* assumption that the lingual gyrus would show activation to the visual stimulus, we extracted beta values of BOLD signal and correlated these with MRS metabolites values.

Statistical analyses were performed with SPSS Statistics Version 24.0 for Windows (IBM, Armonk, NY: IBM Corp.; http://www.spss.com). Shapiro–Wilk Test was used to assess normal distribution of variables. Students *t*‐test or Mann–Whitney *U* test for nonparametric variables were used to compare mean values between groups. Spearman’s Rho was used to analyze correlations between variables. A moderated regression analysis was used to determine if relationships between variables depended on group membership. *P* < 0.05 was considered significant.

## Results

### Demographic and clinical data

The two groups presented no significant differences with regards to mean age (VS patients: 28 ± 6 years and controls: 28 ± 5; ±SD, *P* = 0.8), gender (female:male ratio for VS patients 12:12 and controls 14:10; *P* = 0.6) or handedness (right:left ratio for VS patients 21:3 and controls 23:1; *P* = 0.3). Demographic characteristics and clinical features of the VS patient group can be found in Table 1, as well as the presence of migraine comorbidity. Any medication being taken at the time of the study was at a stable dosing regimen and had no reported effects on VS symptoms.

**Table 1:** Demographic and clinical characteristics of the VS patient group (*n* = 24).

Gender, Age	Age of VS onset	Type of visual static	Presence of additional visual symptoms								MIG	Concomitant medication
			A	T	BF	FL	SL	FLA	NY	PH		
F, 33	#	BW,C,F,T	√	√	√	√	√	√	√	√	√	Multivitamins
M, 28	10	C	√				√					
M, 29	26	BW	√		√	√	√	√		√	√	Levothyroxine, paracetamol PRN
M, 25	19	BW,F,T	√	√	√	√	√	√	√	√	√	
F, 20	#	BW,T	√		√	√	√	√		√	√	Fexofenadine
M, 31	9	BW,F	√		√	√		√	√			Pimecrolimus topical, betamethasone topical
F, 34	#	BW,C,F	√		√		√	√	√		√	
F, 23	#	BW,C	√	√		√	√	√	√		√	Oral contraceptive
F, 21	#	BW,F,T	√	√	√	√	√	√	√	√	√	Paracetamol PRN
M, 27	21	BW	√	√	√	√	√	√		√		
F, 26	26	BW	√	√	√	√	√	√	√	√		Multivitamins, ibuprofen PRN
F, 43	43	BW,F,T	√		√	√	√		√			
F, 34	12	BW		√	√	√	√		√	√	√	
F, 22	#	T	√		√	√	√	√			√	Multivitamins, Paracetamol, Nexplanon
F, 34	31	BW,T	√	√	√	√	√	√	√	√	√	
M, 22	15	F,T			√	√	√		√			
F, 25	#	T	√	√	√	√	√			√		Salbutamol inhaler, multivitamins
F, 26	25	BW,C,F,T	√	√	√		√	√	√	√	√	Paracetamol PRN
M, 22	17	F	√		√	√			√	√	√	Magnesium
M, 31	24	BW	√	√	√	√	√	√		√	√	Paracetamol PRN
M, 35	33	BW	√	√	√	√	√		√	√	√	Levothyroxine, CQ10
M, 19	#	BW				√				√		
M, 29	#	F,T		√	√	√	√					
M, 30	#	BW,F	√	√	√	√	√	√	√		√	Fluticasone nasal spray

#, symptoms present for as long as patient could recall; BW, black and white static; C, coloured static; F, flashing static; T, transparent static; √, symptom reported; A, Afterimages; T, Trailing; BF, blue‐field entoptic phenomena; FL, floaters; SL, self‐light of the eye; FLA, flashes; NY, nyctalopia; PH, Photophobia; MIG, Migraine diagnosis; PRN, pro re nata (i.e. when necessary).

### BOLD fMRI analysis—Within group comparison (Task effect)

A one‐sample *t*‐test within each group for the effect of the visual task (i.e. the “snow‐like” stimulus), showed a large cluster of BOLD activation involving the primary and secondary visual cortices bilaterally. These areas were largely overlapping in patients and controls. In patients, the cluster had the following characteristics: *k* = 8370 voxels, MNI peak voxel coordinates *x* = 10, *y* = −92, *z* = −6. In controls, the cluster had the following characteristics: *k* = 7942 voxels, MNI peak voxel coordinates *x* = 18, *y* = −96, *z* = −8.

**Figure 2:**
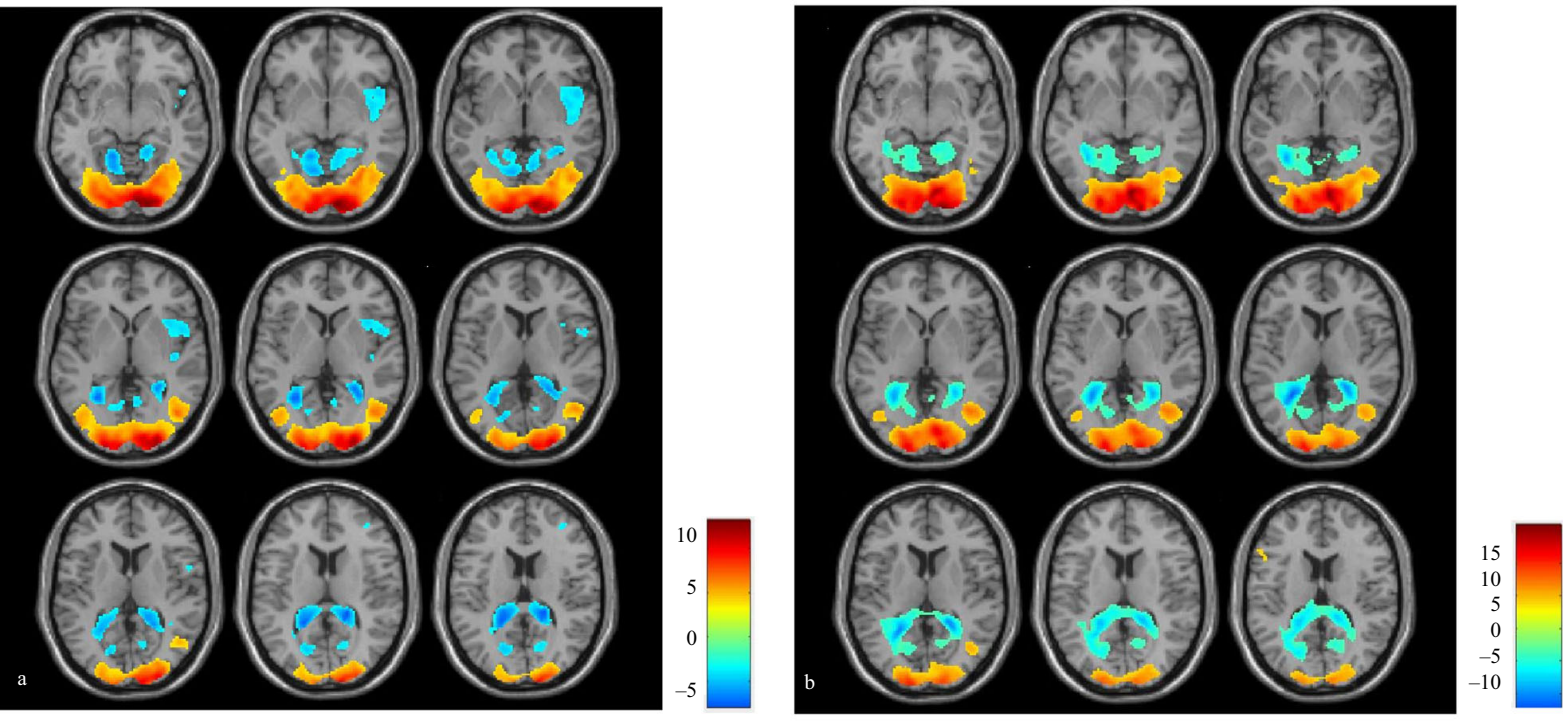
Areas of increased (red/yellow) and decreased (blue/green) BOLD signal in patients (A) and controls (B) when subject to visual ‘snow‐like’ stimulus, respect to baseline. Bars represent *T* values.

Greater responses at baseline than during visual stimulation (deactivations) were found in five clusters in patients and two clusters in controls, involving the periventricular areas bilaterally, as well as, in patients only, the middle frontal gyrus, superior frontal gyrus, frontal eye fields, supramarginal gyrus, frontal operculum, and right insula (Figure 2, Table 2).

**Table 2:** Brain areas of differential BOLD response to visual stimulus respect to baseline, in patients and controls.

Group	Contrast	Brain region	*k*	*P* value	T	Peak coordinates		
		Cluster description				*x*	*y*	*z*
Patients	Activation	Bilateral primary and secondary visual cortices, LG, fusiform gyrus, BA 17‐18‐19	8370	<0.001	13.01	10	−92	−6
Patients	Deactivation	Bilateral periventricular areas, cuneus, precuneus, LG	3862	<0.001	7.73	20	−38	14
		R middle frontal gyrus, R superior frontal gyrus, FEF, BA 8‐9‐10	1013	<0.001	5.66	34	38	28
		L precentral and L post central gyrus, BA 4‐7‐31	297	0.024	4.96	−24	−28	36
		R frontal operculum, R inferior frontal gyrus, R insula, BA 13‐22	685	<0.001	4.91	50	10	0
		R supramarginal gyrus, R inferior parietal lobule, BA 40‐2	288	0.027	4.75	56	−42	44
Controls	Activation	Bilateral primary and secondary visual cortices, lingual and fusiform gyrus, BA 17‐18‐19	7942	<0.001	15.87	18	−96	−8
		L precentral gyrus, L inferior frontal gyrus, BA 45‐6	304	0.003	5.67	−56	10	40
Controls	Deactivation	Bilateral periventricular areas, cuneus, precuneus, LG	6075	<0.001	12.02	−24	−50	8
		L postcentral gyrus	921	<0.001	4.66	−20	−32	54

Cluster coordinates are shown in MNI space with relative T scores and *k* values. An initial voxel threshold of *P* < 0.001 and cluster correction to *P* < 0.05 was applied. R, right; L, left; LG, lingual gyrus; FEF, frontal eye fields; BA, Brodmann area.

### BOLD fMRI analysis—Patients versus controls (Group effect)

**Figure 3:**
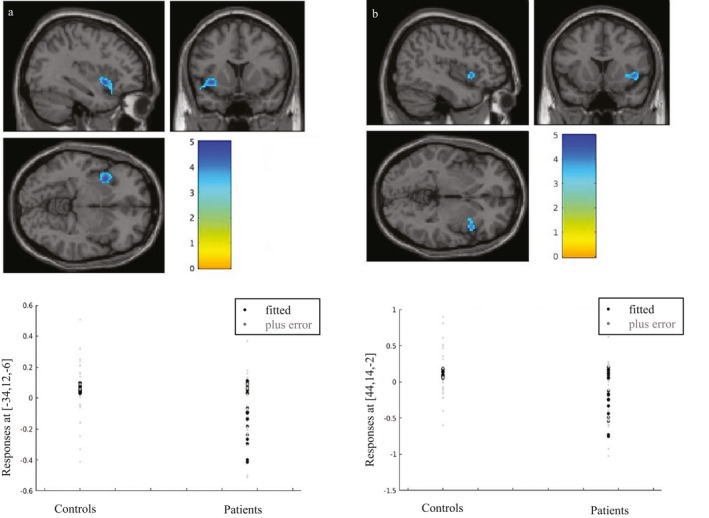
Analysis for BOLD group differences in patients versus controls, in response to visual stimulation (i.e. ‘snow‐like’ stimulus) with respect to baseline. Plots for effects of group for the respective clusters are shown on the lower panel. Bars represent *T*‐values. (A) Whole brain analysis showing a reduction of BOLD signal in VS patients in the left anterior insula (*k* = 291; *P* = 0.025; peak MNI coordinates: *x* = −34, *y* = 12, *z* = −6). (B) SVC with anatomical mask over the right anterior insula, showing a significant reduction of BOLD signal in VS patients (*k* = 100; *P* = 0.003; peak MNI coordinates: *x* = 44, *y* = 14, *z* = −2).

A whole‐brain voxel‐wise analysis comparing patients versus controls, revealed one significant cluster of difference in the BOLD response of patients with respect to controls, located in the left anterior insula (MNI peak voxel coordinates: *x* = −34, *y* = 12, *z* = −6) and involving *k* = 291 voxels. This area is shown in Figure 3A. A cluster could also be seen in the contralateral side; this did not reach significance in the whole‐brain analysis but did for correction within an anatomically derived insula mask (created in the “wfu_pickupatlas”) (MNI peak voxel coordinates: *x* = 44, *y* = 14, *z* = −2; *k* = 100; Figure 3B). When examining the betas and plotting for the effect of group, we found that these bilateral insular clusters reflected a greater deactivation (i.e. greater BOLD activity at baseline than during the visual stimulus) in VS patients compared to controls (Figure 3, lower panel).

There were no significant areas of increased BOLD signal in patients compared to controls.

### MR spectroscopy

**Figure 4:**
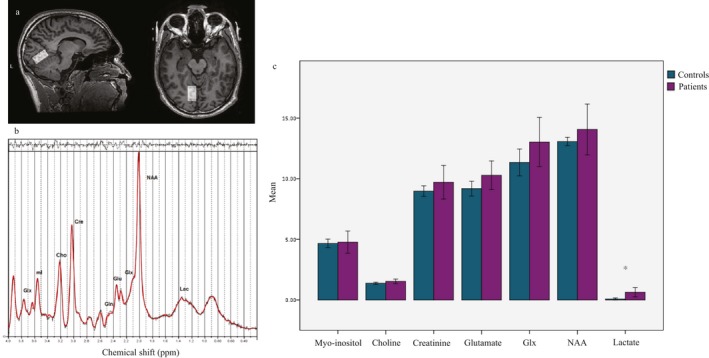
Magnetic resonance spectroscopy results. (A) Example of voxel placement in the right lingual gyrus and (B) example spectrum in one subject. (C) Average metabolite concentrations in visual snow patients versus controls. ^*^Represents significant differences (*P* < 0.001; obtained with Mann‐Whitney U test).

One subject in the control group did not have a spectroscopy scan. Furthermore, one patient had low spectra signal‐to‐noise ratio (7.0) and was excluded from the analysis. The average SNR of the remaining forty‐six spectra was 22.5 (±3.4), whereas average FWHM was 0.05 ± 0.01 ppm (5.77 ± 1.04 Hz). See Figure 4 for location of the voxel and an example spectrum.

When comparing the mean metabolite concentrations of NAA, choline, creatine, myo‐inositol, glutamate, Glx (glutamate + glutamine), and lactate, we found a significant increase in lactate levels in the VS patient group with respect to the control group (0.66 ± 0.9 mmol/L vs. 0.07 ± 0.2 mmol/L; *P* < 0.001; Figure 4). We also found a trend of significance for glutamate differences (10.44 ± 2.8 mmol/L vs. 9.18 ± 1.4 mmol/L; *P* = 0.06; Figure 4).

### Correlation analysis

**Figure 5:**
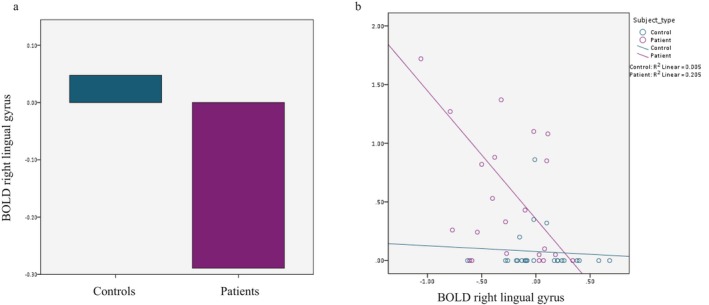
(A) Average BOLD responses in the right lingual gyrus, in VS patients versus controls (−0.29 vs. 0.03; *P* < 0.001). (B) Correlation between lactate concentrations and BOLD beta values in the right lingual gyrus (*P* = 0.004; *r* = −0.42).

When analyzing the extracted beta values for the BOLD response of the anatomically‐derived right lingual gyrus ROI, we found that these values were significantly different between the two groups (average in patients: −0.29 vs. controls: 0.03; *P* < 0.001; Figure 5A).

We then performed a correlation analysis between the BOLD beta values from this ROI and lactate concentration in the MRS voxel across all subjects, finding that there was a significant negative correlation between the two (*P* = 0.004; *r* = −0.42; Figure 5B). A moderated regression to account for group membership further confirmed that lactate concentrations influenced BOLD beta values from the right lingual gyrus (*P* = 0.04), and that this relationship was moderated by being in the VS group (OR = −0.221; 95% C.I.  = −0.432 − 0.011).

## Discussion

Using BOLD‐fMRI we show reduced activations to a visual stimulus in the anterior insular cortices of VS patients compared to healthy subjects. Furthermore, the region of the right lingual cortex has increased lactate concentrations compared to controls, with a negative correlation between lactate concentration and BOLD‐fMRI responses to visual stimulation in the VS group. The data begin to dissect the biological basis of VS syndrome, in terms of both salience networks and a possible localized visual association cortex dysfunction.

### Response to visual stimulation in VS

The bilateral deactivations in the insular cortex could be interpreted in two opposite ways. They could represent a reduction of activity in the insula when the brain is involved in a visual task, or rather they could be due to an increased activation of this region when the brain is at rest and not involved in the processing of an external stimulus. The latter hypothesis certainly is in line with the well‐known role of the insula within the salience network. The anterior insula in particular is essential for selecting information that is relevant for the brain, amongst an immense variety of different stimuli that are constantly being received and processed.^22^ This region then conveys the information deemed significant to other areas of the limbic system.^23^ A dysfunction of this fundamental activity of integration could explain, at least in part, the underlying pathophysiology of VS, where stimuli that should normally be considered irrelevant “pass” a certain salience threshold, finally turning into an apparently normal perception. A further possibility is that, unlike healthy subjects in whom the visual stimulus would capture high levels of attentional and behavioral resources, the “intrinsic” snow is being perceived as more salient by the VS brain than the “external” snow, thus causing the apparent deactivation in the insula when patients are exposed to the outside stimulus.

Our fMRI paradigm also showed an expected BOLD activation of the bilateral primary and secondary visual cortices to the visual stimulation mimicking VS itself, which was strong and sustained in both patients and controls. This confirms that our experimental model of VS constitutes an appropriate tool for further investigation of the condition. Visual cortex activations were accompanied by bilateral deactivations in the occipital horns of the lateral ventricles, which extended to occipital gray matter areas of the cuneus, precuneus, and lingual gyrus. Furthermore, in patients only, we found a decreased BOLD response in several cortical areas, corresponding to the right middle frontal gyrus, superior frontal gyrus, precentral gyrus, frontal eye fields, supramarginal gyrus, frontal operculum, and insula. Aside from the insular region, these deactivations did not survive comparison between groups; nonetheless, they could suggest an abnormal reorganization of attentional networks in VS in response to an external stimulus. The frontal eye fields and supramarginal gyrus in particular are directly involved in the control of visual awareness and visuo‐spatial attention.^24^, ^25^ Conversely the periventricular changes in BOLD signal common to both groups could represent noise, the product of a nonstationarity artifact,^26^ or potentially—given the high *T*‐scores and based on previous studies performed in cats^27^ and humans^28^—be caused by physiological changes in CSF volume in response to rapid changes in visual stimuli.

### Spectroscopy of the lingual gyrus

Our magnetic resonance spectroscopy results showed increased lactate and a trend for increased glutamate in the right lingual gyrus of VS patients. Lactate is a product of anaerobic glycolysis; its brain concentrations can increase in response to pathological increases in energy demand, such as cerebral hypoxia, ischemia, or seizures, and more generally in the case of mitochondrial and metabolic dysfunctions.^29^ Transient increases in lactate levels are also found with ^1^H‐MRS as a physiological response to visual stimulation in healthy subjects^30^, ^31^; this occurs because of a temporary excess of glycolysis over respiration in the cortex.^32^ Previous studies have found similar lactate alterations in the visual cortex of migraine with aura patients when subject to photic stimulation.^33^, ^34^ In this respect, however, it is important to note that our protocol involved spectroscopy acquisitions at rest, thus suggesting a permanently less efficient metabolism in VS. This, combined with [^18^F]‐FDG PET data,^11^ allows us to hypothesize that the pathologically continuous perception of visual symptoms could be caused by an abnormal involvement of the lingual gyrus, that is metabolically hyperactivated in VS patients.

The trend for glutamate increase in the lingual gyrus that we found in VS subjects is also of note. As glutamate represents the major excitatory neurotransmitter in the brain, an increase in its concentrations could strengthen the hypothesis of hyperexcitability. Furthermore, lactate concentrations can rise in response to glutamate increase as a protective mechanism against excitotoxicity,^35^ and this could partially explain the association between these two metabolites in our subjects. Given the difficulties in distinguishing glutamate from glutamine peaks reliably at lower field strengths,^36^, ^37^ however, these results should be considered with caution.

### Impaired visual cortex metabolism in VS

By combining our MRS analysis of the lingual gyrus with the BOLD responses from the same cortical area, we were able to study both the function and metabolism of the associative visual cortex in VS. The presence of a correlation between decreased BOLD responses and increased lactate levels suggests that the discussed localized disturbance in extrastriate anaerobic metabolism, is in turn causing a decreased capacity for the processing of regular visual stimuli, presented in the form of a snow simulation in our experiment. In other words, it is possible that if the associative visual cortex is continuously hyperactivated in the processing of an irrelevant, but nonetheless continuously perceived, visual symptom, this preactivation could in turn cause a decrease in the metabolic reserve necessary to respond to a physiological external stimulus. This mechanism is particularly useful in explaining photophobia, a key element of the VS syndrome, although it unfortunately falls short of determining the *primum movens* of VS pathophysiology, that is, the “generation” of the snow illusion itself.

A similar mechanism—albeit from a different starting point, as explained below—has been described in migraine, where a decreased habituation, thought to be caused by high preactivation levels of sensory cortices, causes an anomalous response to sensory stimuli. This common dysfunction could indeed represent the link between the conditions of migraine and VS, that are highly comorbid and share many clinical aspects.^11^

### Limitations

A limitation for this study lies in the known difficulties with MRS lactate detection at 3 Tesla fields.^38^ However, given that the shortcomings for this technique are typically characterized by false‐negative results, it is reasonable to conclude that the increase in lactate seen in our population represents a real finding.

Furthermore, a high percentage of our VS population had a concomitant migraine diagnosis. This comorbidity confounds the investigation of VS syndrome, limiting generalizability of results away from patients who do not have co‐existent migraine. On the other hand, specifically selecting VS patients without migraine would result in a selection bias, creating a patient group not representative of the full condition.

Several fMRI studies have investigated BOLD responses to a visual stimulus in migraineurs, both with and without aura, finding increased activation in the primary and associative visual cortex and higher order visual areas, both during the interictal or premonitory period^39^, ^40^, ^41^ and during visually triggered attacks.^42^ Given that these results are opposite to the ones presented here, we hypothesize that our main findings are in fact due to the visual snow condition alone.

### Conclusion

In conclusion, our BOLD‐fMRI and MRS data build on previous findings in VS, and show that at least part of the underlying biology of the syndrome could be characterized by dysfunctions in the visual association cortex as well as the salience network.

## Conflict of Interest

The authors declare no conflict of interest relevant for this manuscript.

## Data Availability

The data that support the findings of this study are available from the corresponding author.
